# Perspectives on Precision Medicine in Chronic Lymphocytic Leukemia: Targeting Recurrent Mutations—NOTCH1, SF3B1, MYD88, BIRC3

**DOI:** 10.3390/jcm10163735

**Published:** 2021-08-22

**Authors:** Maciej Putowski, Krzysztof Giannopoulos

**Affiliations:** 1 Department of Experimental Hematooncology, Medical University of Lublin, 20-093 Lublin, Poland; krzysztof.giannopoulos@gmail.com; 2 Department of Hematology, St. John’s Cancer Center, 20-090 Lublin, Poland

**Keywords:** chronic lymphocytic leukemia, NOTCH1, SF3B1, MYD88, BIRC3

## Abstract

Chronic lymphocytic leukemia (CLL) is highly heterogeneous, with extremely variable clinical course. The clinical heterogeneity of CLL reflects differences in the biology of the disease, including chromosomal alterations, specific immunophenotypic patterns and serum markers. The application of next-generation sequencing techniques has demonstrated the high genetic and epigenetic heterogeneity in CLL. The novel mutations could be pharmacologically targeted for individualized approach in some of the CLL patients. Potential neurogenic locus notch homolog protein 1 (NOTCH1) signalling targeting mechanisms in CLL include secretase inhibitors and specific antibodies to block NOTCH ligand/receptor interactions. In vitro studies characterizing the effect of the splicing inhibitors resulted in increased apoptosis of CLL cells regardless of splicing factor 3B subunit 1 (SF3B1) status. Several therapeutic strategies have been also proposed to directly or indirectly inhibit the toll-like receptor/myeloid differentiation primary response gene 88 (TLR/MyD88) pathway. Another potential approach is targeting nuclear factor kappa-light-chain-enhancer of activated B cells (NF-κB) and inhibition of this prosurvival pathway. Newly discovered mutations and their signalling pathways play key roles in the course of the disease. This opens new opportunities in the management and treatment of CLL.

## 1. Introduction

Chronic lymphocytic leukemia (CLL) is the most common adult leukemia in western countries. CLL is classified as a lymphoproliferative disorder characterized by accumulation of clonal B lymphocytes showing a characteristic immunophenotype (i.e., CD19+, CD20weak, CD23+) in the peripheral blood, bone marrow, lymph nodes and spleen [1]. The clinical course of CLL is highly heterogeneous, with the majority of patients following an indolent course with no or delayed treatment, while others experience aggressive disease with survival of few months [2]. The clinical heterogeneity of CLL reflects differences in the biology of the disease, including chromosomal alterations, gene mutations, specific immunophenotypic patterns and serum markers [3]. Despite significant progress in its management, CLL remains an incurable disease [4].

In the 1990s, cytogenetic abnormalities assessed by conventional karyotyping in CLL patients were found to be associated with shorter survival in a study by Juliusson et al. [5]. According to the cytogenetic model presented in 2000 by Döhner et al., CLL patients may be stratified based on the four most common chromosomal alterations (deletion of 13q14, deletion of 11q22-23, deletion of 17p12 and trisomy 12). Chromosomal alterations, assessed by FISH molecular karyotyping, have been detected in 82% of patients affected by CLL [6]. Of these, deletion of 13q14, occurring in 50–60% of cases, is the most frequent genetic lesion of CLL [6,7].

CLL cells express the B cell receptor (BCR) on their surface, which is a membrane bound form of the immunoglobulin molecule (IG). Surface IG expression plays a key role in survival and functioning of normal B cells, and also of many B cell lymphoproliferative disorders [2]. In 1999, it was independently reported by the Hamblin/Stevenson and Chiorazzi groups that somatic hypermutations present in rearranged immunoglobulin heavy variable (*IGHV*) genes in CLL predict a different clinical course [8,9]. Somatic mutations of *IGHV* gene (defined as <98% identity to the germline *IGHV* gene) occur in approximately half of CLL cases and are usually characteristic by more favourable prognosis. This contrasts with patients with unmutated CLL (*IGHV* gene sequences with a germline homology of 98% or higher), who have a more aggressive disease with worse prognosis [8,9].

Notably, 30% of CLL patients express quasi-identical BCR IG, the so-called “stereotyped” receptors, and can be classified into subsets defined by distinctive sequence motifs within the IG variable heavy complementarity-determining region 3 (VH CDR3). Stereotyped subsets are characterized by similar biological features, and similar disease course and outcome [10,11,12]. It has been suggested that subset classification can supersede general division into CLL patients with mutated and unmutated *IGHV* [13].

In recent years, the application of next-generation sequencing (NGS) techniques has demonstrated the high genetic and epigenetic heterogeneity in CLL [14]. The novel, previously unknown, mutations which were revealed include neurogenic locus notch homolog protein 1 (*NOTCH1*), splicing factor 3B subunit 1 (*SF3B1*), tumor protein p53 (*TP53*), myeloid differentiation primary response gene 88 (*MYD88*), ataxia telangiectasia mutated (*ATM*), baculoviral IAP repeat containing 3 (*BIRC3*) and chromodomain-helicase-DNA-binding protein 2 (*CHD2*) [2,14,15,16,17]. These recurrent somatic mutations were found to be involved in key cellular pathways, such as DNA damage response, cell cycle regulation, apoptosis, RNA metabolism, NOTCH signalling, nuclear factor (NF)-kB signalling, chromatin remodelling and inflammatory BCR pathways [2,14,15,16,17]. Among these mutations, *TP53, NOTCH1, SF3B1,* and *BIRC3* have been established as prognostic factors for the course of CLL and proposed to be incorporated in CLL prognostic scales [18,19,20]. The International Workshop on Chronic Lymphocytic Leukemia published in 2018 guidelines which included assessment of TP53 mutation in routine practice. As of today, evaluation of others molecular targets such as NOTCH1, SF3B1, and BIRC3 mutations is not an element of the routine prognostic work up in CLL. However, for clinical trials only, molecular testing is recommended before treating a patient on protocol [21].

Over the past decade, the implementation of the Bruton’s tyrosine kinase (BTK), phosphoinositide 3-kinase (PI3K) inhibitors and venetoclax overturned CLL treatment and replaced chemotherapy-based treatments for most CLL patients [22]. The consequent advances in understanding the clinical and biological heterogeneity of CLL and the development of new targeted therapies are leading us to an individualized, personalized approach [4].

## 2. *NOTCH1* Mutation

The *NOTCH1* gene encodes a member of the NOTCH family of proteins. The NOTCH1 receptor acts as a ligand-activated transcription factor that directly transduces extracellular signals leading to changes in gene expression in the nucleus, including *MYC*, *TP53* and molecules of the NF-kB pathway [23,24,25]. The majority of *NOTCH1* mutations disrupt the PEST domain of the protein, which is responsible for the proteasomal degradation of the of NOTCH1 receptor, resulting in a truncated, constantly active protein [7]. Additionally, recurrent mutations in the noncoding 3′UTR of *NOTCH1* and rare, loss-of-function mutations in FBXW7, a ubiquitin ligase implicated in NOTCH1 turnover, have also been identified [15,17]. NOTCH1 signalling activation was confirmed to play a role in resistance to apoptosis and increased CLL cell survival [26,27,28]. In addition, recent studies revealed the alternative non-mutational mechanisms of NOTCH1 activation in CLL, indicating that constitutive activation of the NOTCH1 pathway in this leukemia is more frequent than previously estimated by the incidence of genetic lesions [29]. Clinically, *NOTCH1*-mutated patients have been associated with more aggressive clinical presentations of the disease such as being chemorefractory and having a high risk of disease progression toward transformation into Richter syndrome. Affecting up to 10–15% of CLL patients at diagnosis, *NOTCH1* mutations are an independent predictor of survival in CLL [27]. *NOTCH1* mutations are more frequently detected in patients harbouring trisomy 12 and cases with unmutated *IGHV* genes [27]. CLL patients with *NOTCH1* mutations do not benefit from rituximab-combining therapies, which may be related to lower levels of CD20 expression in *NOTCH1* mutated cases [30,31], while a longer progression-free survival was demonstrated when treated with alemtuzumab [32]. Among anti-CD20 antibody therapies, obinutuzumab with higher efficacy compared to rituximab, has shown to overcome the refractoriness in CLL patients carrying *NOTCH1* mutation [33].

**Figure 1:**
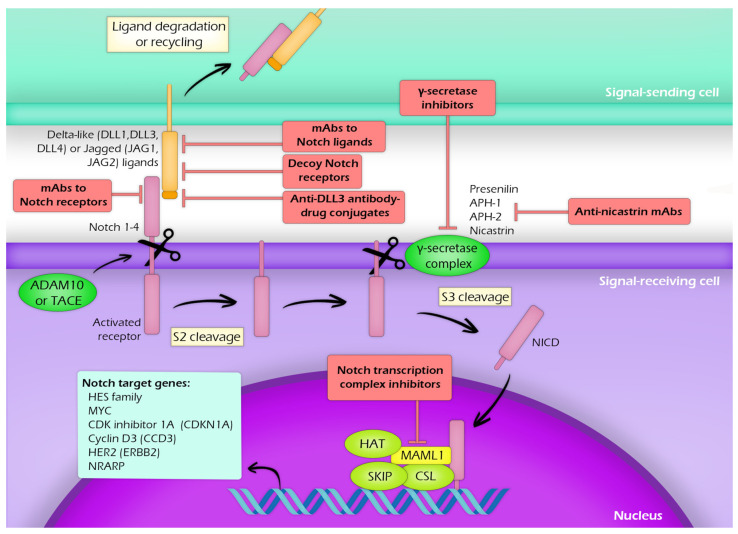
*NOTCH1* signaling pathway and potential inhibitory strategies.

The canonical NOTCH signalling pathway and potential pharmacological inhibitors are shown in Figure 1. Moreover, therapeutic strategy involving the administration of non-coding RNAs has emerged as a possible novel approach of targeting NOTCH signaling [34].

For this reason, targeting NOTCH signalling has emerged as a promising therapeutic strategy for CLL. Potential NOTCH1 signalling targeting mechanisms in CLL include secretase inhibitors (GSIs) and specific antibodies anti-NOTCH1 receptor. Lopez-Guerra, et al. reported the antitumor effect of using the GSI PF-03084014 in combination with fludarabine in CLL cells carrying *NOTCH1* mutations [35]. Additionally, the PF-03084014 and fludarabine combination impairs angiogenesis and CXCL12-induced responses associated with tumoral migration and invasion [23,35]. Moreover, GSIs were confirmed to have a therapeutic effect in T-cell acute lymphoblastic leukemia (T-ALL), where more than 50% of patients harbor *NOTCH1* activating mutations [36]. The main limitations of the use of GSIs in clinical practice include non-selectivity and gastrointestinal toxicity [36]. Nevertheless, GSIs are still being explored in clinical trials with the aim of optimizing the dose regimen and reducing side effects through distinct formulations [37,38,39].

The other approach of targeting the NOTCH1 pathway is the use of antibodies to block NOTCH ligand/receptor interactions. Among different NOTCH ligands, delta-like ligands DLL4 and DLL1 play crucial roles in CLL, with DLL4 being the most potent stimulator of NOTCH signalling in *NOTCH1*-mutated CLL cases [40]. The specific antibodies against the specific NOTCH receptors have been already developed [41,42]. OMP-52M51, an anti-human NOTCH1 monoclonal antibody, was studied in xenograft models of T-ALL and demonstrated promising antitumor efficacy [43]. OMP-52M51 inhibits DLL4-induced Notch stimulation and cell proliferation and also reverses the Notch-induced *MYC*, *CCND1*, and *NPM1* gene expression [44]. The study conducted by Lopez-Guerra et al. [40], suggested that DLL4 expressed by the tumor microenvironment activates NOTCH signaling in CLL. Based on this result, the protumour processes in CLL could be disrupted by specific NOTCH targeting.

## 3. *SF3B1* Mutation

SF3B1 is a core component of the spliceosome, a complex of five small nuclear ribonucleoproteins RNA (snRNPs), the splicing machinery involved in the process of RNA editing through the removal of introns in protein-encoding genes [45]. The product of the *SF3B1* gene is considered as an essential component that catalyses the removal of intronic sequences and ligates the exons into mature, functional mRNA [46]. The *SF3B1* mutations lead to deregulation of the mRNA splicing process due to intron retention, exon skipping and abnormal splice site insertion. The functional consequences of *SF3B1* mutations are associated with alteration of multiple cellular functions, including the DNA damage response, telomere maintenance and NOTCH pathway signalling [47].

Multiple bacterial-derived products and their synthetic analogs display antitumor activities and bind tightly to components of the spliceosome [48]. Together with other genes encoding splicing factors, *SF3B1* is one of the most highly mutated genes in various hematological malignancies, including CLL. For this reason, drugs targeting the spliceosome are being sought. Among the first identified compounds displaying cytotoxic effects in tumor cell lines by an arrest in the G1 and G2/M phases of the cell cycle were FR901463, FR901464 and FR901465 (obtained from Pseudomonas spp.), GEX1 and pladienolides (Streptomyces spp.). Subsequently, synthetic analogs with improved stability and solubility were discovered such as spliceostatin A (SSA), meayamycin, sudemycins, E7107, and most recently, H3B-8800 [48,49,50,51,52].

The preclinical models have confirmed that splicing inhibitors are selectively lethal to tumor cells [53]. In a mouse model of leukemia, treatment with E7107 resulted in prolonged survival and reduced leukemic burden [54]. The oral analog of E7107, H3B-8800, inhibits cell growth in human AML cell lines and induces apoptosis preferentially in cells carrying the K700E *SF3B1* mutation [53].

In CLL, in vitro studies characterizing the effect of exposure to FD-895, pladienolide B and SSA resulted in increased apoptosis of CLL cells regardless of *SF3B1* status [15,55,56]. Similarly, promising effects both in vitro and in a xenograft model were observed with exposure to sudemycins, where even low doses of sudemycins provoked apoptosis of CLL cells. Sudemycin has also shown an enhanced antitumor response in combination with ibrutinib in CLL, which may be related to the alteration of BTK signaling [57].

Subsequently, human phase I clinical trials of E7107 were conducted, initially in patients with advanced solid tumours. The therapeutic effect of stabilization of tumor growth was achieved, however the study was discontinued due to optical and gastrointestinal toxicity [58]. The other compound, a selective small molecule *SF3B1* modulator, H3B-8800, has been studied in patients with myelodysplastic syndromes, acute myeloid leukemia and chronic myelomonocytic leukemia and found to have a good safety profile and tolerability. However, neither objective complete nor partial response, except laboratory and clinical improvement, was observed [59]. The actual, ongoing clinical trials involving newly designed molecules targeting mutations/pathways of NOTCH, SF3B, MYD88/TLR, BIRC3 are presented in Table 1.

## 4. *MYD88* Mutation

MYD88 is a cytoplasmic adaptor protein that plays an essential role in the innate and adaptive immune response. The protein encoded by the *MYD88* gene is required for signal transmission by toll-like receptors (TLRs), except TLR3, and receptors for IL-1 as well as IL-18 [60,61]. After stimulation, *MYD88* activates the NF-kB pathway and the mitogen-activated protein kinase (MAPK) pathway by forming a signalling complex that consists of various intermediary proteins, such as IL-1R-associated kinases (IRAKs) and tumor necrosis factor receptor-associated factors (TRAFs), most notably TRAF6 [62,63].

A single-nucleotide change (c.794T.C), is the most common mutation of *MYD88* resulting in a change from leucine into proline at position 265 (L265P) [64]. The functional effects of the *MYD88* mutation include increased NF-κB activity, increased JAK-STAT3 signalling and production of pro-inflammatory cytokines such as IL-6, IL-10, and IFN-β, as well as enhanced survival of lymphoma cells [64,65]. The *MYD88* mutation has been found at high frequencies in cutaneous diffuse large B cell lymphoma (DLBCL; 69%), primary central nervous system lymphoma (38%), Waldenström’s macroglobulinemia (WM; almost 100% of cases) and activated B cell DLBCL (39%), indicating its role in the pathogenesis of lymphoid neoplasias [65,66]. In CLL, *MYD88* mutations occur at a variable frequency of 1.5% to 10% and are found predominantly in patients with mutated *IGHV* and chromosome 13q deletions, both of which are associated with lower-risk disease [18,67,68,69]. However, multiple studies in CLL have shown the *MYD88* mutation to have a favourable prognosis [70] or no association with the course of disease [18,67], whereas others have shown the *MYD88* mutation to be associated with an unfavourable prognosis [68]. Thus, the functional role of *MYD88* in CLL has not been fully elucidated.

Several therapeutic strategies have been proposed to inhibit the TLR/MYD88 pathway directly or indirectly by targeting IRAK1 and IRAK4 in the myddosome-complex, TAK1 in downstream signalling, BTK in the BCR pathway, TLR9 in the My-T-BCR supercomplex, and components of the concurrently activated PI3K/AKT/mTOR and HCK pathways [63,71,72]. Although the BTK is not a *MYD88* (L265P)-specific target and is not directly involved with the MYD88-derived protein complex, inhibition of BTK has been widely studied and revealed as the most successful therapy in CLL [71]. Nevertheless, MYD88-derived peptides can induce T-cell responses, which supports the idea of a potential T-cell receptor-based immunotherapy [63].

As for IRAK inhibitors, several studies with promising results identified two compounds, ND-2158 and ND-2110, which blocked IRAK4 in vitro and in xenografts with human DLBCL cell lines [72,73]. A recent study conducted by Giménez et al. [74] has shown similar effects in CLL in both in vitro and in vivo models. The dose-dependent antitumor effect confirmed the importance of the MYD88/TLR pathway in CLL and suggests IRAK4 may be a therapeutic target for this disease. Additionally, the combination of an IRAK4 inhibitor with ibrutinib or venetoclax demonstrated superior antitumor activity [74,75]. The preclinical tests of IMO-8400, an oligonucleotide specifically designed to inhibit ligand activation of TLR7/8/9 have shown inhibition of cell signalling and reduction of tumor growth [76]. Two phase I/II clinical trials (NCT02252146 and NCT02363439) in MYD88-positive DLBCL and WM patients have been performed, with the results showing that IMO-8400 is well tolerated [71]. More research is required to implement MYD88-derived treatment in the future.

## 5. *BIRC3* Mutation

The *BIRC3* gene encodes a member of the inhibitor of apoptosis (IAP) family of proteins, c-IAP2. BIRC3 has been identified as a negative regulator of the MAP3K14 serine-threonine kinase and the alternative noncanonical NF-κB signaling pathway [77]. *BIRC3* mutations, accounting for 2% to 10% of CLL patients, are associated with high-risk disease, shorter progression-free survival (PFS) and overall survival (OS) [78,79]. There is an association between the *BIRC3* mutations and unmutated *IGHV* genes, trisomy 12 and deletion of 11q in CLL patients [78]. Functionally, *BIRC3* mutation led to constitutive NFkB activation, which may contribute to the mechanism of resistance to treatment in leukemia and tumuor growth through downregulation of the TP53 protein via MDM2 [80]. CLL patients harbouring *BIRC3* mutations more commonly develop chemorefractoriness characterized by fludarabine resistance [78]. Additionally, BIRC3 mutations incidence is rare at the time of diagnosis, but in fludarabine-refractory patients is increased to approximately 25% [19]. The CLL14 phase 3 clinical showed a shorter progression-free survival in the chlorambucil-obinutuzumab arm versus venetoclax-obinutuzumab, reinforcing the role of BIRC3 mutations as a biomarker of chemorefractoriness [81]. In these patients, other treatments, such as cyclin-dependent kinase inhibitor, BTK inhibitor, B cell lymphoma 2 inhibitor, alemtuzumab and corticosteroids might be considered [82].

BIRC3 disrupting mutations in CLL lead to constitutive NF-κB pathway activation promoting proliferation and survival [83]. BIRC3 has no relevant role in physical inhibition of caspases resulting in direct inhibition of apoptosis but regulates NF-κB signalling [84]. Targeting NF-κB and inhibition of this prosurvival pathway represent a possible strategy for the treatment of CLL patients [85]. In vitro studies have shown that low or absent *BIRC3* expression was associated with increased survival of CLL cells [86]. Silencing Map3K14 (also known as NIK–NF-kB-inducing kinase) decreased the levels of NFκB, which was followed by reduced viability of BIRC3-mutated cells [87]. Physiologically, BIRC3 catalyses MAP3K14 protein ubiquitination leading to proteasomal degradation [88]. With this regard, targeting Map3K14—the centrally activating kinase—remains under investigation [88]. Two NIK inhibitors (AM-0216 and AM-0650) demonstrated activity in myeloma cells with NIK-dependent activation of NF-kB [89]. However, the potential of NF-κB inhibitors in targeting the *BIRC3* abnormalities requires further validated studies.

## 6. Conclusions

The mutational landscape in CLL is changing the understanding and management of the disease. Newly discovered mutations and their signalling pathways play key roles in the course of the disease. The novel biomarkers should be considered in terms of prognosis as well as predictive factor. Nowadays, assessment of molecular abnormalities may contribute to the choice of treatment strategy. *NOTCH1, SF3B1* and *BIRC3* mutations are negative prognostic markers in CLL. Moreover, rituximab should be avoided in *NOTCH1* mutated patients, while *BIRC3* mutation is highlighted as a marker of chemorefractoriness. Ongoing studies directly affecting molecular pathways may open new opportunities in the management and treatment of CLL. Further research on the efficacy and safety of novel drugs is necessary to develop targeted, personalized therapy in CLL patients.

## Figures and Tables

**Table 1:** Ongoing clinical trials associated with targeting mutations/pathways in haematological malignancies.

Gene/Pathway	Drug/Mechanism of Action	Phase	Conditions	Identifier
NOTCH	CB-103/pan-NOTCH inhibitor	I/II	Metastatic Solid Tumours, Haematological Malignancies	NCT03422679
SF3B	JNJ-64619178/Inhibitor of PRMT5	I	Neoplasms, Solid Tumor, NHL, MDS	NCT03573310
	H3B-8800/Spliceosome inhibitor	I	MDS, AML, CMML	NCT02841540
MYD88 /TLR	CA-4948/IRAK4 kinase inhibitor	I/II	NHL, WM/LPL, CLL/SLL	NCT03328078
	CA-4948/IRAK4 kinase inhibitor	I/II	AML, MDS	NCT04278768
	SD-101/TLR9 agonist	I	NHL	NCT03410901
	SD-101/TLR9 agonist	I/II	B cell lymphoma	NCT02927964
	Poly-ICLC/TLR3 agonist	I/II	Low grade lymphoma	NCT01976585
BIRC3	ASTX660/cIAP1 and XIAP inhibitor	I	AML	NCT04155580
	ASTX660/cIAP1 and XIAP inhibitor	I/II	T-cell lymphoma	NCT04362007
	ASTX660/cIAP1 and XIAP inhibitor	I/II	Lymphomas	NCT02503423

Table includes ongoing studies registered at ClinicalTrials.gov (Accessed date 10 August 2021). Abbreviations: AML: acute myeloid leukemia; CLL: chronic lymphocytic leukemia; CMML: chronic myelomonocytic leukemia; IRAK4: IL-1R-associated kinase 4; MDS: myelodysplastic syndromes; MYD88/TLR: myeloid differentiation primary response gene 88 / toll-like receptor; NHL: non-Hodgkin lymphoma; NOTCH: neurogenic locus notch homolog protein; Poly-ICLC: Polyinosinic-polycytidylic acid, and poly-L-lysine; PRMT5: protein arginine methyltransferase 5; SF3B: splicing factor 3B subunit; SLL: small lymphocytic lymphoma; TLR: toll-like receptor; WM/LPL: macroglobulinemia/lymphoplasmacytic lymphoma.

## Data Availability

Not applicable.

## Abbreviations

APH-1/2: anterior pharynx-defective-1/2; CSL: CBF1/Su(H)/Lag-1; DLL: delta-like ligand; HAT: histone acetyltransferase; HES: hairy and enhancer of split-1; JAG: Jagged ligand; mAb: monoclonal antibody; MAML1: Mastermind-like 1; NICD: Notch intracellular domain; NRARP: Notch-regulated ankyrin-repeated protein; SKIP: ski-interacting protein; TACE, TNF-α-converting enzyme (also known as ADAM17).
